# Rediscovery of PF-3845 as a new chemical scaffold inhibiting phenylalanyl-tRNA synthetase in *Mycobacterium tuberculosis*

**DOI:** 10.1016/j.jbc.2021.100257

**Published:** 2021-01-08

**Authors:** Heng Wang, Min Xu, Curtis A. Engelhart, Xi Zhang, Baohua Yan, Miaomiao Pan, Yuanyuan Xu, Shilong Fan, Renhe Liu, Lan Xu, Lan Hua, Dirk Schnappinger, Shawn Chen

**Affiliations:** 1 Global Health Drug Discovery Institute, Haidian, Beijing, China; 2 Department of Microbiology and Immunology, Weill Cornell Medical College, New York, New York, USA; 3 Center of Protein Science Facility, Tsinghua University, Beijing, China

**Keywords:** phenylalanyl-tRNA synthetase (PheRS), high-throughput screening, crystallography, inhibition mechanism, *Mycobacterium tuberculosis*, aaRSs, aminoacyl-tRNA synthetases, CV, column volume, DMSO, dimethyl sulfoxide, FAAH, fatty acid amide hydrolase, FARS, human phenylalanyl-tRNA synthetase, HTS, high-throughput screening, MESG, 2-amino-6-mercapto-7-methylpurine ribonucleoside, MIC, minimum concentration required to inhibit >90% growth of WT Mtb H37Rv, Phe, phenylalanine, PheRS, bacterial phenylalanyl-tRNA synthetase, PNPase, purine nucleoside phosphorylase, PPase, pyrophosphatase, PPi, pyrophosphate, TB, tuberculosis

## Abstract

*Mycobacterium tuberculosis* (Mtb) remains the deadliest pathogenic bacteria worldwide. The search for new antibiotics to treat drug-sensitive as well as drug-resistant tuberculosis has become a priority. The essential enzyme phenylalanyl-tRNA synthetase (PheRS) is an antibacterial drug target because of the large differences between bacterial and human PheRS counterparts. In a high-throughput screening of 2148 bioactive compounds, PF-3845, which is a known inhibitor of human fatty acid amide hydrolase, was identified inhibiting Mtb PheRS at *K*_*i*_ ∼ 0.73 ± 0.06 μM. The inhibition mechanism was studied with enzyme kinetics, protein structural modeling, and crystallography, in comparison to a PheRS inhibitor of the noted phenyl–thiazolylurea–sulfonamide class. The 2.3-Å crystal structure of Mtb PheRS in complex with PF-3845 revealed its novel binding mode, in which a trifluoromethyl–pyridinylphenyl group occupies the phenylalanine pocket, whereas a piperidine–piperazine urea group binds into the ATP pocket through an interaction network enforced by a sulfate ion. It represents the first non-nucleoside bisubstrate competitive inhibitor of bacterial PheRS. PF-3845 inhibits the *in vitro* growth of Mtb H37Rv at ∼24 μM, and the potency of PF-3845 increased against an engineered strain Mtb pheS–FDAS, suggesting on target activity in mycobacterial whole cells. PF-3845 does not inhibit human cytoplasmic or mitochondrial PheRS in biochemical assay, which can be explained from the crystal structures. Further medicinal chemistry efforts focused on the piperidine–piperazine urea moiety may result in the identification of a selective antibacterial lead compound.

Protein synthesis is the cellular process targeted by many commercial antibiotics. It has always been a focal point of modern antibacterial drug discovery (1). Aminoacyl-tRNA synthetases (aaRSs), a family of ∼20 essential enzymes, ligate amino acids to the corresponding tRNAs that decode messenger RNA to produce protein at the translating macromolecular ribosome (2). Inhibition of bacterial aaRS blocks the translation and ultimately shuts down protein synthesis, which is crucial for pathogens to survive in host or inside host cells (3, 4). Between a bacterial aaRS protein and its human counterpart, either the large sequence difference or small variation of key residues in the catalytic core explains the high selectivity of successful and promising aaRS inhibitor drugs, as exemplified by the mupirocin used for the treatment of staphylococcal infection (4). Tuberculosis (TB) caused by the single agent *Mycobacterium tuberculosis* (Mtb) has surpassed AIDS/HIV, becoming a leading infectious disease worldwide (https://www.who.int/news-room/fact-sheets/detail/tuberculosis). Most TB drugs were discovered in the past century and are losing efficacy because of the resistance inevitably arisen in bacteria. New chemical scaffold with novel inhibition mechanism against Mtb is being actively sought. An oxaborole compound GSK3036656 that inhibits Mtb leucyl-tRNA synthetase is currently undergoing clinical trial (5).

Bacterial phenylalanyl-tRNA synthetase (PheRS) is a member of class II aaRS based on the active site topology (6). A functional PheRS is typically made of two heterodimers (αβ)_2_, with a whole molecular weight around 250 kD. In Mtb H37Rv genome, two consecutive essential genes, *Rv1649* and *Rv1650* (*pheST*), encode the protein subunits (7). Like other aaRSs, PheRS catalyzes the formation of phenylalanyl-tRNA^Phe^ in two steps: (1) activation of phenylalanine (Phe) by hydrolyzing ATP to form Phe-AMP and pyrophosphate (PPi) and (2) subsequent transfer of Phe to the 2′-OH group of adenosine ribose at the 3′-terminal of tRNA^Phe^ and simultaneous release of AMP. In addition to the synthetic site on the α subunit, PheRS has a dedicated domain on the β subunit that can hydrolyze improperly charged tRNA, for example, tyrosinyl-tRNA^Phe^, to maintain the fidelity of aminoacylation and translation (8). From a drug discovery standing point, PheRS synthetic activity that involves binding of the three substrates and the editing activity could all be targetable. PheRS should also allow specific binding of compounds that allosterically regulate the enzymatic activities because of its size, molecular mechanism, and other miscellaneous functions (9). PheRS was a major antibacterial target in a number of screening programs (10, 11, 12, 13, 14, 15, 16, 17). Bacterial PheRS-specific hits such as phenyl–thiazolylurea–sulfonamides have emerged from synthetic compound library. To the best of our knowledge, most of them were not for TB drug except one that identified a fungal natural product isopatulin able to inhibit the growth of Mtb strain H37Rv (18). Recently, PheRS from *Plasmodium falciparum*, which is another pathogen of global health concern, has become genetically and chemically validated target for antiparasitics (19). Potent PheRS inhibitors with novel mechanisms at the molecular and cellular levels were discovered (20), prompting us to initiate a hit discovery campaign targeting Mtb PheRS with new assays and diversity compound libraries, such as recent collection of pharmacologically relevant small molecules (21).

In this work, Mtb PheRS enzyme was enzymatically characterized and analyzed with a synthesized reference compound GDI05-001. High-throughput screening (HTS) assay was adopted to screen a Selleck-2148 library against the PheRS. One screening hit, PF-3845, was confirmed to inhibit Mtb PheRS with *K*_i_ ∼ 0.73 μM. It is a well-developed inhibitor of human fatty acid amide hydrolase (FAAH). Its minimum concentration required to inhibit >90% growth of WT H37Rv (MIC) is ∼24 μM, and the antibiotic activity increased against an engineered strain Mtb pheS–FDAS, which expresses PheS contained a C-terminal degradation tag Flag-DAS. The mechanism of inhibition was studied with enzyme kinetics and crystal structures. PF-3845 was shown to be a non-nucleoside bisubstrate competitive inhibitor of the PheRS. The results will aid future medicinal chemistry effort to improve the potency, reduce the toxicity, and develop combination therapy with TB drugs.

## Results

### Overexpression, purification, and characterization of Mtb PheRS

In Mtb H37Rv genome, *pheST* genes encode the α and β subunits of PheRS, respectively. The deduced amino acid sequence of PheS is ∼16 to 30% identical to human cytoplasmic hFARS1α or mitochondrial hFARS2. The mitochondrial PheRS does not have a β subunit. The identity of PheT *versus* hFARS1β is below 12%. Three conserved signature motifs of class II aaRS are identified in Mtb PheS sequence (Fig. S1). The ∼3.5 kb *pheST* was synthesized based on the DNA sequences in GenBank. After sequencing confirmation, the DNA was cloned for heterologous expression in *Escherichia coli*. Several constructs were made for copurification of the heterodimeric protein. The best one, pET30a-Mtb PheRS-CHis, under optimized fermentation conditions, gave a yield of ∼1 to 2 mg/l, with the highest purity possible after optimized purification steps (Fig. S2). We also constructed, overexpressed, and partially purified an Mtb tRNA_GAA_^Phe^ from *E. coli* (see Experimental procedures section). It was reported that more than ∼90% of the enriched and partially purified tRNA fraction could be the overexpressed tRNA species (22, 23). We used standard aminoacylation assay that detects the charging of ^14^C-labeled Phe onto tRNA^Phe^ to confirm the purified protein and tRNA were indeed functional together. Michaelis–Menten kinetic parameters of Mtb PheRS were measured with the tRNA aminoacylation assay and shown in Figure S3. The *K*_m_'s and *k*_cat_'s generally agree with previous reports on Mtbs and other bacterial PheRSs although some discrepancies are noted. For example, we and others found the *K*_m_ with regard to Phe at ∼68.0 ± 6.6 μM, but this is much higher than the ∼1 to 7 μM of other bacterial PheRSs (14, 15, 24, 25). The parameters are subject to revision in further research of PheRS biology and aromatic amino acid metabolism.

### Assay development and screening for inhibitors of enzymatic activity

**Figure 1:**
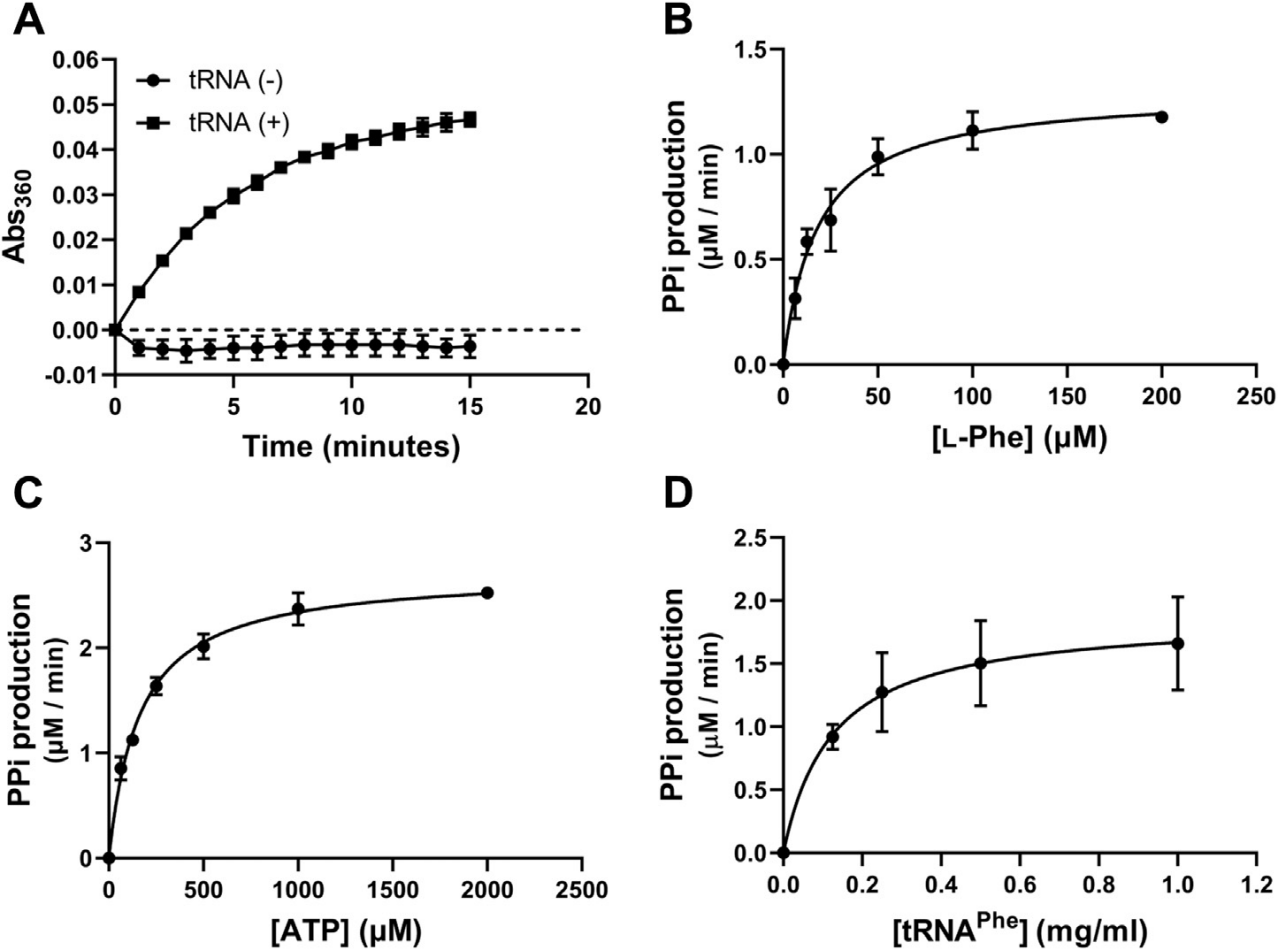
**Mtb PheRS pyrophosphate (PPi) production is dependent on tRNA**^**Phe**^**and the Michaelis–Menten kinetics.** The assay is a PPi production assay (see Experimental procedures section) measuring the continuous release of phosphate in the presence of pyrophosphatase that hydrolyzes PPi. *A*, PPi generation in the PheRS reaction with or without tRNA^Phe^. *B*–*D*, *K*_m_ determination of Mtb PheRS with respect to l-Phe, ATP, or tRNA^Phe^. The data were collected from separate experiments with three sample replicates and are presented as mean ± SD (*n* = 3). Mtb, *Mycobacterium tuberculosis*; PheRS, phenylalanyl-tRNA synthetase.

Nonradioactive biochemical assays were adopted for screening for PheRS inhibitor and the subsequent analysis of mode of inhibition. In a continuous spectrophotometric assay (26), the PPi production is coupled to generation of phosphate by inorganic pyrophosphatase (PPase); the phosphate then serves as a substrate in a purine nucleoside phosphorylase (PNPase)–catalyzed reaction to cleave nucleoside compound 2-amino-6-mercapto-7-methylpurine ribonucleoside (MESG) into ribose-1-phosphate and base 2-amino-6-mercapto-7-methylpurine that has absorbance at 360 nm. When optimizing the so-called PPi production assay, we noticed that the A_360_ readout is tRNA dependent as expected, meaning no PPi was released in the absence of tRNA (Fig. 1*A*). PheRS concentration at 100 nM per reaction gave a linear response in the first 6 min of the progression. By measuring the initial velocity of PPi production, the apparent *K*_m, ATP_ with regard to ATP was found to be 162.50 ± 11.62 μM, *K*_m, Phe_ 18.10 ± 2.44 μM, and *K*_m, tRNA_ 0.13 ± 0.05 μg/ml (Fig. 1, *B*–*D*). These *K*_m_'s were used as references in the following screening and hit confirmation.

To screen compound library, we used a commercial kit Kinase-Glo, which measures the remaining amount of ATP in a PheRS reaction. Readout is luminescent signal generated by luciferase-catalyzed oxidation of luciferin in the presence of the ATP. The readout is thus inversely correlated with the amount of PheRS activity. Because ATP at 1 μM to initiate the PheRS reaction is far below its *K*_*m*_, the assay is presumably biased toward ATP-competitive inhibitors (27). We optimized the assay in 384-well HTS format and determined the amount of PheRS per reaction (Fig. S4). Reaction endpoint was chosen to be at the first 2 h. A known bacterial PheRS inhibitor (11) was synthesized as reference compound, named GDI05-001 (NMR and MS data in Supporting Information). Its IC_50_ against Mtb PheRS was determined to be 1.72 ± 0.13 μM.

**Figure 2:**
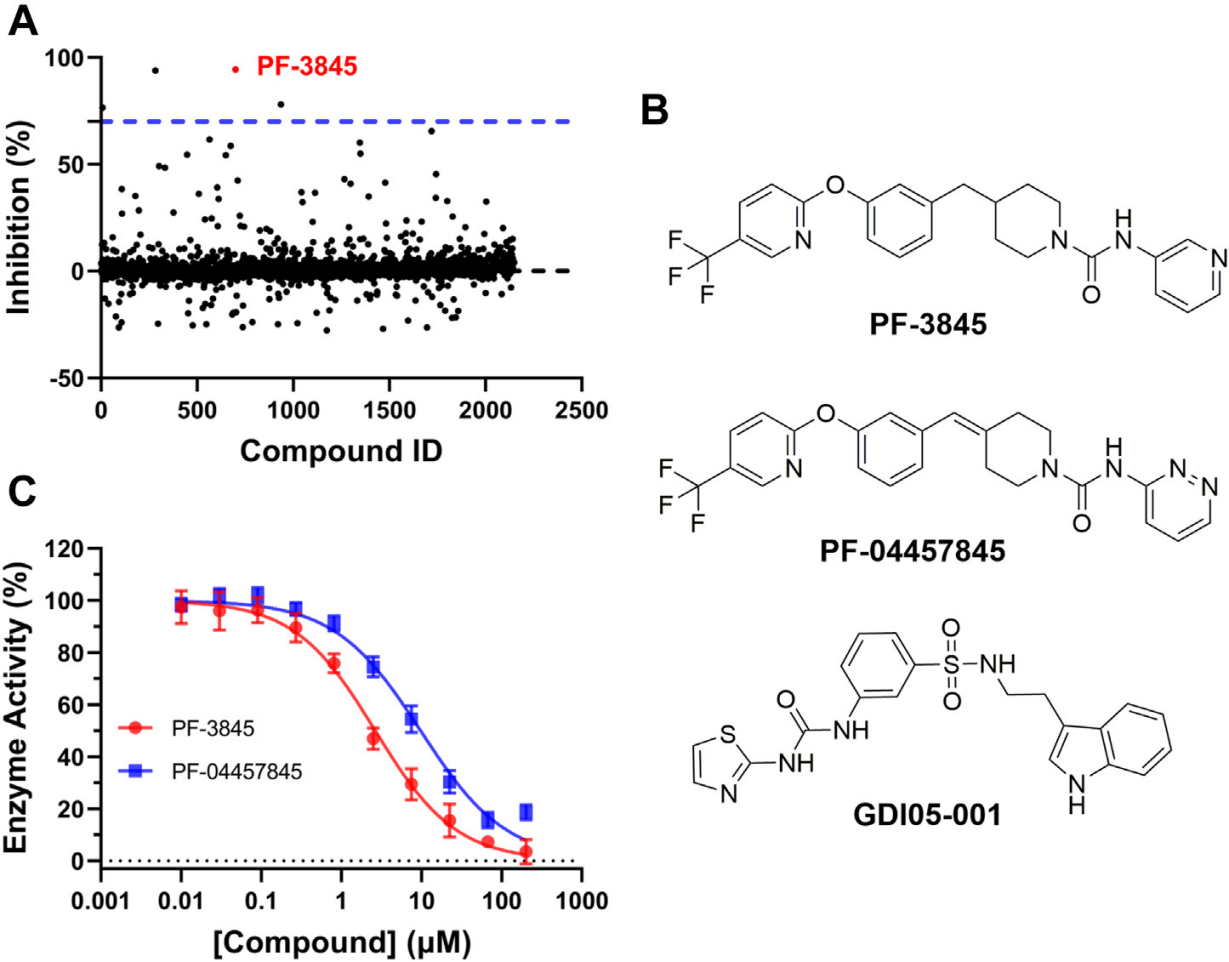
**Result of high-throughput screening with Selleck-2148 library and hit confirmation.***A*, summary plot of the high-throughput screening result. *B*, structures of PF-3845, a purchased analog PF-04457845, and reference compound GDI05-001. *C*, IC_50_ of PF-3845 and 004457845 against *Mycobacterium tuberculosis* phenylalanyl-tRNA synthetase by Kinase-Glo assay, presented as mean ± SD (*n* = 3). See supporting information for IC_50_s determined by other assays.

A Selleck Bioactive Collection purchased before 2018 contains 2148 small molecules with validated biological and pharmacological activities. The library was screened in seven plates, which showed mean robust *Z*′ at 0.756 to 0.862. When the cutoff was set to 70% inhibition compared with no-enzyme control reaction, four initial hits were identified (Fig. S5), giving a hit rate ∼0.19% (Fig. 2*A*). A top hit PF-3845 is reportedly a potent covalent inhibitor of human enzyme FAAH with *K*_*i*_ calculated to be 0.23 ± 0.03 μM (28), and many analogous compounds are in public database. PF-3845 and an analog PF-04457845 were repurchased (Fig. 2*B*). The IC_50_s of the two compounds were determined with the two assays and other similar assays (29) (Fig. S6). By the Kinase-Glo assay, the IC_50_ of PF-3845 was 2.65 ± 0.19 μM (hillslope −0.86 ± 0.05) and the IC_50_ of PF-04457845 was 9.76 ± 0.73 μM (hillslope −0.81 ± 0.05) (Fig. 2*C*). PF-3845, at concentrations up to 20 μM, did not inhibit the activities of PPase, PNPase, and luciferase that were used in the coupled reactions (Fig. S7). The inhibition was observed only when Mtb PheRS was present in the coupled reactions. Thus, we identified investigational drug PF-3845 as a new PheRS inhibitor. PF-3845 apparently has a scaffold different from previously known PheRS inhibitors.

### Analysis of the mode of inhibition by kinetics, crystallography, and modeling

**Figure 3:**
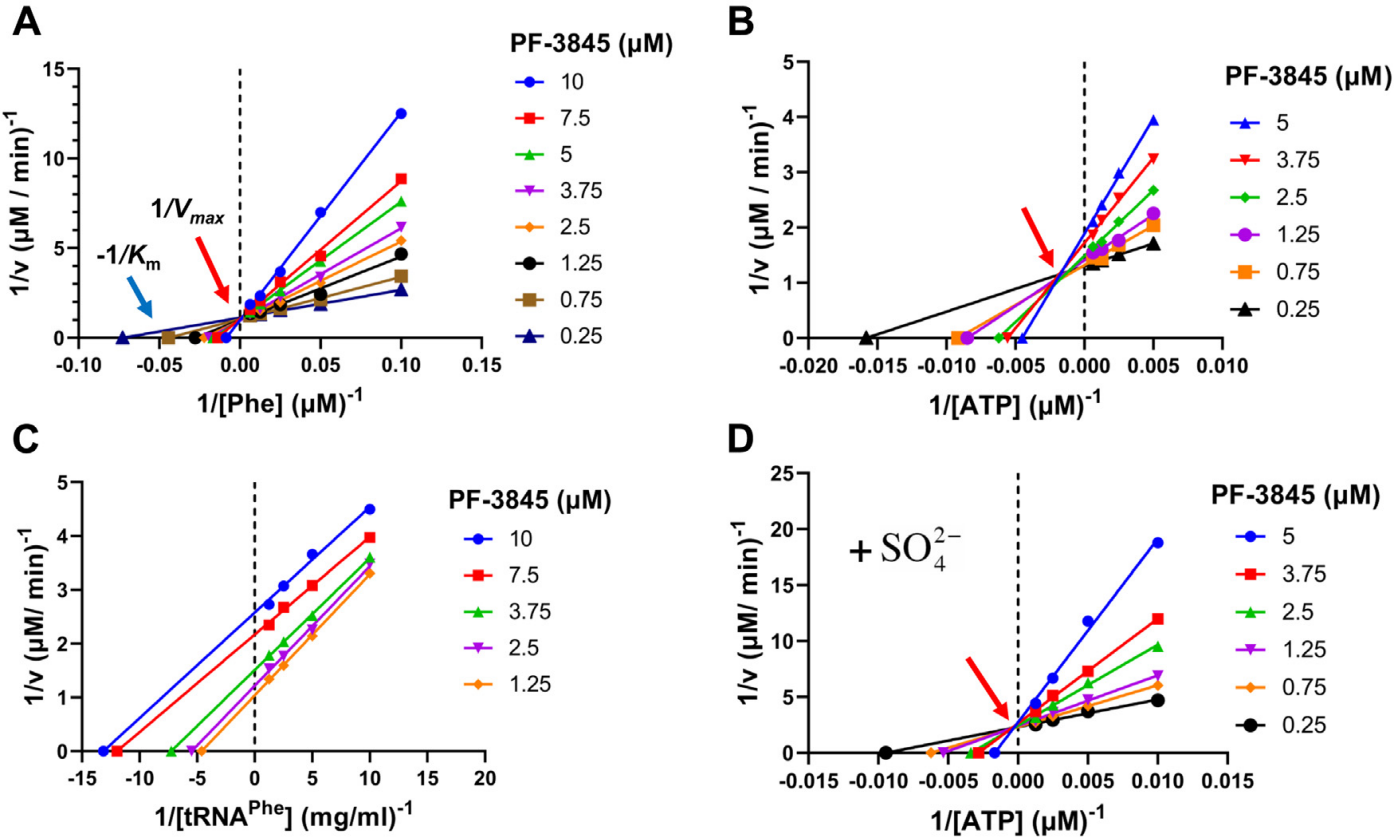
**Mode of inhibition of PF-3845, analyzed by Lineweaver–Burke plot with respect to one of the substrates****l****-Phe (*A*), ATP (*B*), tRNA (*C*), and ATP with SO**_**4**_^**2−**^**(*D*) added into the buffer, using the pyrophosphate production assay.***A*, competitive inhibition mode regarding l-Phe. *B*, mixed inhibition mode regarding ATP. *C*, uncompetitive inhibition mode regarding tRNA^Phe^. *D*, competitive inhibition mode regarding ATP, in the presence of 5 mM (NH_4_)_2_SO_4_.

The mode of inhibition of PF-3845 was analyzed with enzyme kinetics in comparison with GDI05-001. PPi production assay was used to record the progress of PheRS reaction setups, which had excessive amounts of two substrates but varying concentration of a third substrate at various inhibitor concentrations. Each dataset was fit into Michaelis–Menten equation; the resulting Lineweaver–Burk plot of a set of experiments with regard to the third substrate was examined for a pattern of competitive, noncompetitive, uncompetitive, or mixed inhibition mode. Both GDI05-001 and PF-3845 are clearly Phe competitive inhibitors of PheRS as the presence of inhibitor only affected *K*_*m*_ but not *V*_max_ (Fig. 3*A*). In the Phe-competitive mode, *K*_*i*_ of GDI05-001 is 0.20 ± 0.01 μM and *K*_*i*_ of PF-3845 is 1.70 ± 0.11 μM, determined with the PPi production assay. It was reported that GDI05-001 was noncompetitive regarding ATP (11), and we had similar observation. In contrast, PF-3845 is more likely in a mixed inhibition mode with regard to ATP over a range of PF-3845 concentrations (Fig. 3*B*). PF-3845 is an uncompetitive inhibitor with regard to tRNA^Phe^ (Fig. 3*C*).

**Figure 4:**
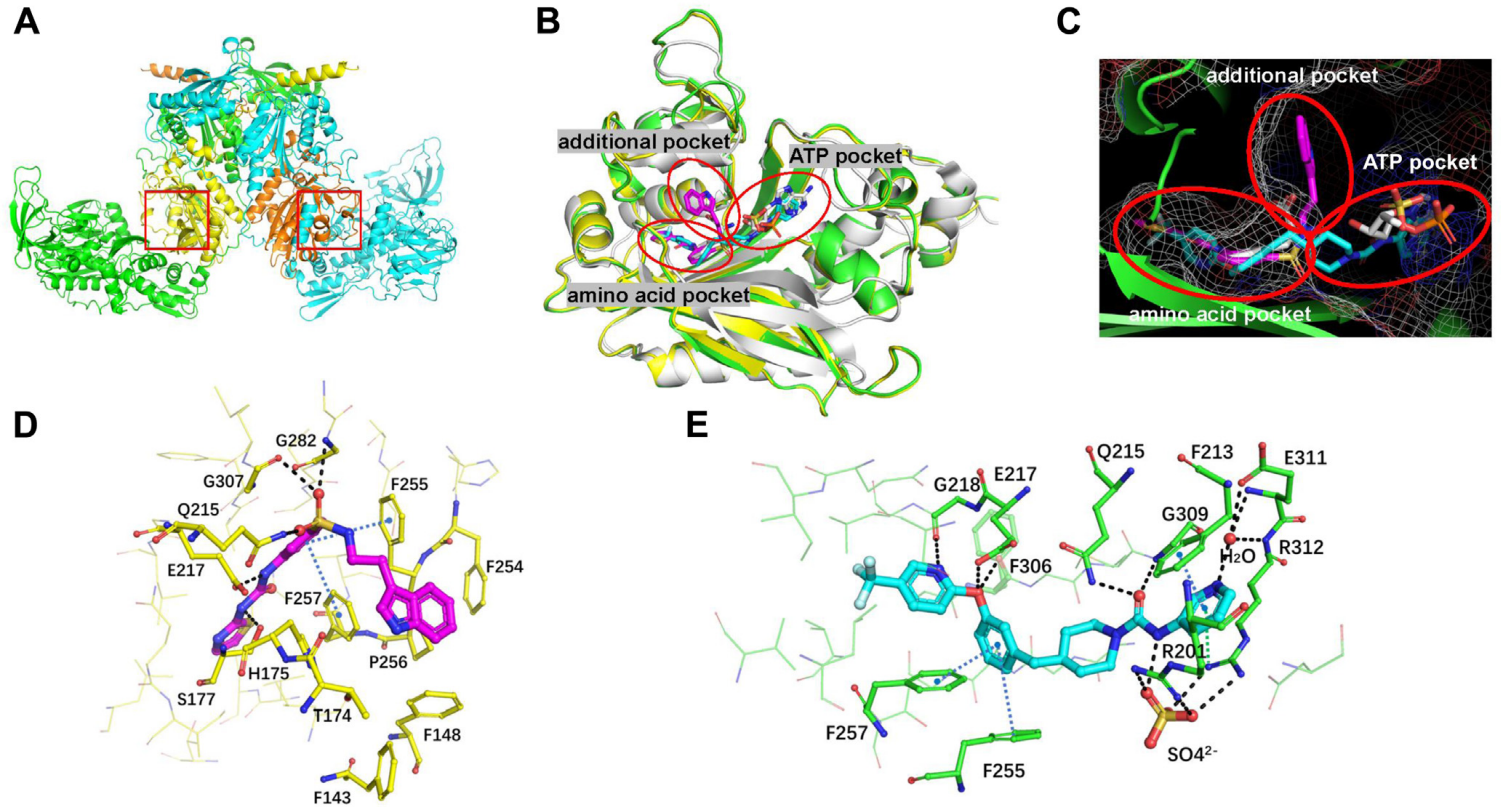
**Overall structures of Mtb PheRS and three binding pockets of the catalytic site.***A*, overall structure of apo Mtb PheRS. The α subunits are shown in *yellow* and *orange*, and the β subunits are shown in *green* and *cyan*. The catalytic sites of the α subunits are indicated by *red boxes*. *B*, structure superposition of the α subunits of Mtb PheRS–GDI05-001, Mtb PheRS–PF-3845, and EcPheRS–Phe-AMP (Protein Data Bank: 3PCO). Mtb PheRS–GDI05-001 is shown in *yellow* with ligand in *magenta*, Mtb PheRS-PF-3845 is shown in *green* with ligand in *cyan*, EcPheRS–Phe-AMP is shown in *white*. Three binding pockets of the catalytic site are indicated by *circle*. *C*, three binding pockets of the catalytic site are shown in mesh based on the structure of Mtb PheRS–PF-3845. *D*, the interaction network between GDI05-001 and Mtb PheRS. *E*, the interaction network between PF-3845 and Mtb PheRS. Hydrogen bonds are shown in *black dash*, π–π stackings are shown in *blue dash*, and π–cation interaction is shown in *green dash*. Mtb, *Mycobacterium tuberculosis*; PheRS, phenylalanyl-tRNA synthetase.

Crystallographic effort was made to first solve the structures of Mtb PheRS apo protein and its complex with GDI05-001 at 2.83 and 2.71 Å, respectively. Like other bacterial PheRS structures, Mtb PheRS is an (αβ)_2_ heterotetramer. The α subunit contains catalytic site, and β subunit contains editing site (Fig. 4*A*). Except for the N-terminal 74 residues of α subunit, all other residues were constructed. The N-terminal region of *Thermophilus thermus* PheRS was reported to form a coiled-coil domain, playing a critical role in interaction with tRNA (30). It was flexible and invisible in the solved Mtb PheRS when no tRNA was included. The binding mode of GDI05-001 was illustrated by cocrystallization (Fig. 4*B*). When looking into the catalytic site of the α subunit, we found that besides the amino acid (Phe) pocket and ATP pocket, there is an additional pocket between the two pockets (Fig. 4*C*). The key residues forming the additional pocket are mainly hydrophobic, including F143, F148, A154, H175, F254, F255, P256, and F257. GDI05-001 occupies the Phe pocket and the additional pocket, whereas the ATP pocket is empty (Fig. 4, *C* and *D*). The phenylsulfonamide group of GDI05-001 extends much deeper into the amino acid pocket than a Phe could. In this pocket, strong π–π stackings can be seen between the phenyl rings of GDI05-001 and F255/F257. Three hydrogen bonds are formed between the urea group of GDI05-001 and the side chains of E217 and S177. In addition, the sulfonamide oxygen forms interactions with the side chain of Q215 and the main chain of G282 and G307. The indole moiety of GDI05-001 is bended almost vertically to the phenylsulfonamide and extends into the additional pocket, which has strong hydrophobic interaction with the protein. Compared with the apo form, two major differences were observed. First, in the apo form structure, the side chain of F257 has two alternative conformations with one conformation that occupies the deeper amino acid pocket, and the side chain of Q180 also stretches toward the pocket. While in the liganded structure, F257 adapts only one rigid conformation, which occupies out of the pocket. Together with the rotation of Q180, they constitute a much deeper amino acid pocket of the complex than the apo form (Fig. S8). Second, the loop 148 to 160 undergoes a conformational change upon GDI05-001 binding and has much higher B factors than the apo structure, indicating it is more flexible when GDI05-001 binds (Fig. S9).

On the other hand, preliminary analysis using a deep learning–based docking platform Orbital showed that PF-3845 might have two major putative binding conformations among the top-ranking poses (Fig. S10). In one conformation, PF-3845 is bended to interact with the additional pocket outside Phe pocket by π–π interactions. Another possibility is that PF-3845 could extend deep into the ATP pocket. Further work is needed to validate which binding mode is dominated.

### Cocrystal structure of PheRS-PF3845 and the role of a sulfate ion in the ligand binding

**Figure 5:**
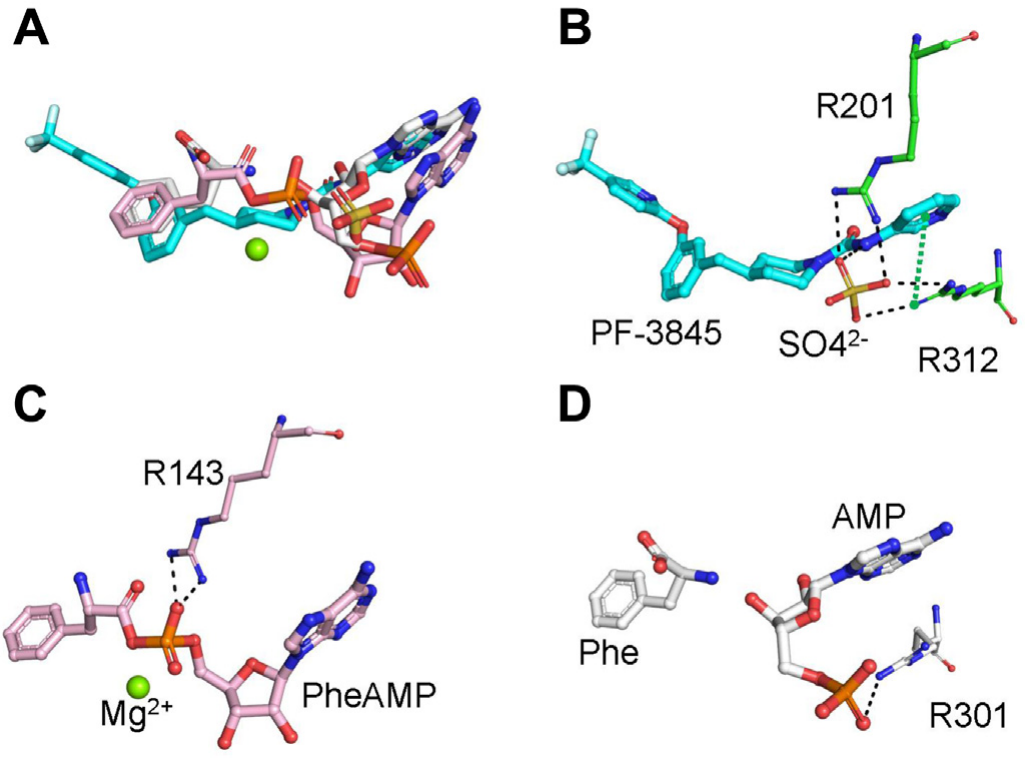
**Sulfate ion stabilizes the interaction between PF-3845 and Mtb PheRS.***A*, structural comparison of the bound ligands of Mtb PheRS–PF-3845, hFARS2–PheAMP (Protein Data Bank: 3CMQ), and EcPheRS–Phe-AMP (Protein Data Bank: 3PCO). *B*, the SO_4_^2−^ interacts with the amide linker of PF-3845 and R201/R312 in Mtb PheRS–PF-3845. *C*, the PheAMP interacts with R143 in hFARS2–PheAMP structure. *D*, the AMP interacts with R301 in EcPheRS–Phe-AMP structure. Mtb, *Mycobacterium tuberculosis*; PheRS, phenylalanyl-tRNA synthetase.

To illustrate the binding mode of PF-3845, we obtained the cocrystal structure of Mtb PheRS in complex with PF-3845 at a resolution of 2.3 Å. Initial Fo–Fc map of PF-3845 (along with GDI05-001) is presented in Figure S11. PF-3845 binds clearly in the amino acid pocket as well as the ATP pocket, whereas the additional pocket is empty (Fig. 4*C*). An extensive hydrophilic and hydrophobic interaction network in two pockets can be found between PF-3845 and Mtb PheRS. Like the phenylsulfonamide group of GDI05-001, the 4-trifluoromethyl-2-pyridinyl group and phenyl ring of PF-3845 inserts deeply into the amino acid pocket. Two strong π–π stackings also can be seen between the phenyl rings of PF-3845 and F255/F257 residues of the PheRS (Fig. 4*E*). A hydrogen bond is formed between the amide of the trifluoromethylpyridinyl group and the main chain oxygen of G218. Two more hydrogen bonds are formed between the oxygen linker and the side chain of E217 and the main chain of F306. Unlike GDI05-001, the other end of PF-3845, including the 3-aminopyridine and piperidine groups, completely inserts into the ATP pocket (Fig. 4, *C* and *E*). The carbonyl group of PF-3845 forms a hydrogen bond with the main chain amide of G309. The amide of 3-aminopyridine forms a water-mediated interaction with the side chain of E311 and the main chain of E311/R312. The 3-aminopyridine of PF-3845 also forms a strong π–π stacking with F213 and a π–cation interaction with R312. Surprisingly, a sulfate ion (SO_4_^2−^) is found near PF-3845 to bridge the interaction between the amide linker of PF-3845 and R201/R312 of the PheRS. Compared with the apo form, the side chain of R312 in the complex rotates nearly 90° to get closer to the compound. The SO_4_^2−^ most likely stabilizes R312, which in turn forms the π–cation interaction with the 3-aminopyridine. When this is compared with two other structures, hFARS2 complexed with Phe–AMP and *E. coli* PheRS (EcPheRS) complexed with Phe and AMP, the SO_4_^2−^ occupies an approximate space of AMP phosphate group in either case (Fig. 5). In the hFARS2–PheAMP cocrystal structure (31), the PheAMP forms hydrogen bond with R143 that is corresponding to R201 in Mtb PheRS. In the EcPheRS–AMP cocrystal structure, the AMP forms hydrogen bond with R301 corresponding to R312 in Mtb PheRS. Overall, the SO_4_^2−^ here engages R201 and R312 in the ATP pocket and enables multiple interactions to facilitate PF-3845 binding into the ATP pocket of Mtb PheRS. Although SO_4_^2−^ has been found at the active site of several other aaRS structures, none of them contributes to the binding of a ligand as in the case of PF-3845 (32, 33).

To confirm the role of the SO_4_^2−^, we reperformed and analyzed the PF-3845 inhibition kinetics with 5 mM (NH_4_)_2_SO_4_ in the PheRS enzyme reaction. This time, PF-3845 clearly acted as an ATP competitive inhibitor (Fig. 3*D*), with the *K*_*i*_ calculated to be 0.73 ± 0.055 μM, which is lower than *K*_*i*_ 1.08 ± 0.245 μM with regard to ATP when SO_4_^2−^ was absent (Fig. 3*B*). The experiment demonstrated that sulfate ion increased the binding of PF-3845, making it a true bisubstrate competitive inhibitor of Mtb PheRS.

### Cellular activity and target engagement of PF-3845

**Figure 6:**
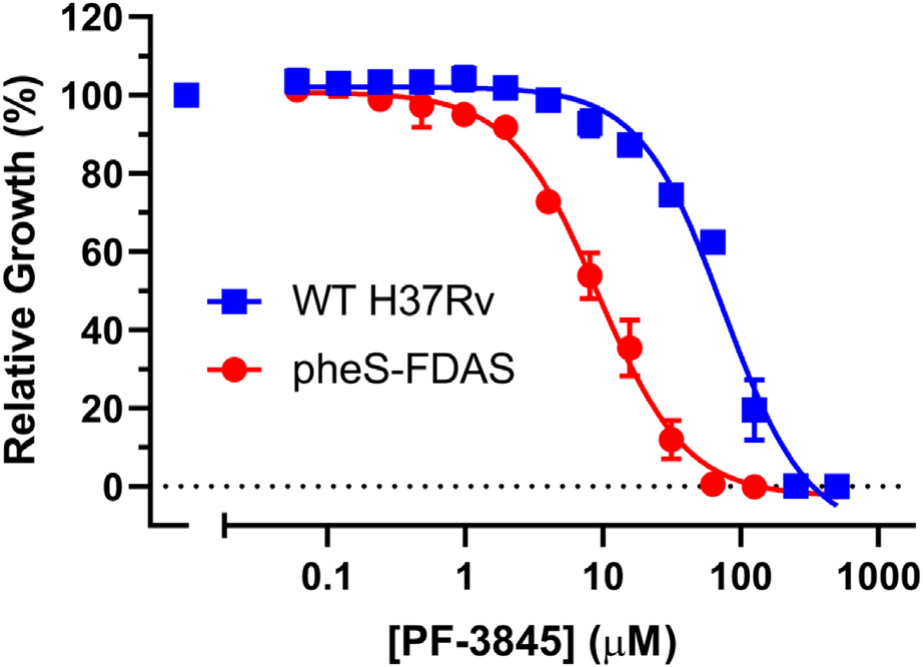
**Susceptibility of WT H37Rv (*blue squares*) and pheS–FDAS (*red circles*) to PF-3845.** Flag- and DAS-tagging PheS expressed in an engineered strain pheS–FDAS derived from WT *Mycobacterium tuberculosis* H37Rv caused the minimum inhibitory concentration of PF-3845 to shift down 5.5-fold compared with WT H37Rv. Results are representative of two independent replicates. Data points shown are the mean ± SD of each dose, tested in triplicate.

The MIC of PF-3845 against Mtb H37R_v_ measured by a microplate alamarBlue assay (34) was ∼23.87 μM (*n* = 3) compared with the MIC of GDI05-001 at 145 μM. It indicates that PF-3845 has better permeability into Mtb cells than GDI05-001 even though its *K*_*i*_ to PheRS target is ninefold higher. The cytotoxicity of PF-3845 or PF-04457845 (CC_50_), shown as 50% inhibition of the proliferation of mammalian cell lines, ranges from 16.9 to 25.8 μM (Fig. S12), which is not surprising for a drug originally discovered as a human protein inhibitor. Next, we set to analyze if PF-3845 can engage PheRS target in Mtb H37R_v_. For this, we used Mtb pheS–FDAS in which *pheS* was tagged to encode a C-terminal degradation tag. The MIC of PF-3845, measured by an absorbance at 580 nm, shifted down approximately 5.5-fold from 220 μM in WT H37Rv to 40 μM in the pheS–FDAS mutant, suggesting that PF-3845 can inhibit PheRS in Mtb (Fig. 6).

## Discussion

GDI05-001 belongs to the phenyl–thiazolylurea–sulfonamide class of PheRS inhibitors originally discovered in 2004 by researchers at Bayer Healthcare (11). Ten years later, an AstraZeneca pharmaceutical research group obtained the cocrystals of *Pseudomonas aeruginosa* PheRS in complex with GDI05-001 and identified an auxiliary pocket that is an extension of the Phe-binding pocket (15). It was argued that the auxiliary pocket is a liability for drug discovery because compound binding in the Phe pocket of bacterial PheRS leads to high screening hit rates, resistance frequencies, and elevated plasma protein binding. Further attempt to improve the physicochemical properties of inhibitors by exploring the hydrophilic ATP-binding pocket resulted in very limited success. In this work, by a small-scale screening with a commercial library of 2184 bioactive compounds, we identified a completely different chemical scaffold, PF-3845, as a submicromolar inhibitor of Mtb PheRS. PF-3845 binds to Mtb PheRS in a way distinctive from GDI05-001. Most strikingly, PF-3845 extends into the ATP pocket of PheRS with the help of an inorganic sulfate ion. It represents the first non-nucleoside bisubstrate adenylation inhibitor of PheRS. Based on the 2.3-Å cocrystal structure, the trifluoromethylpyridinyl group on the west side of PF-3845 could be minimized to reduce its binding to the auxiliary pocket, whereas the *N*-pyridine-carboxamide on the east is elaborated (Fig. 2*B*). This type of modification would overcome the shortfalls of the chemical scaffolds Bayer and AstraZeneca had previously pursued, turning up opportunities for design of TB drug candidate as well as new anti–Gram-negative lead. As the PF-04457845 has already shown a fantastic pharmacokinetic profile in rats and dogs as well as *in vitro* assays with human blood (35), other compounds derived from PF-3845 could also be promising clinical candidate for TB treatment.

Although the piperidine–piperazine urea group of PF-3845 occupies the ATP pocket, it does not bind as deep as nonhydrolyzable phenylalanyl-adenylate analog (PheOH-AMP) or Phe-AMP (36, 37). The latter forms multiple hydrogen bonds with a loop region. Two hydrogen bonds formed between PheRS and PF-3845 are through water or sulfate in buffer. The sulfate plays an important role in forming an interaction network, including electrostatic, cationic–π, and hydrogen bonds that altogether hold the piperidine carbamate inside the ATP pocket. Taking advantage of the available cocrystal complex structure, further optimization can be done rationally. For example, it could gain entropic contribution to the binding affinity if the compound is modified to replace the bridging water. Regarding the sulfate ion, one possible strategy is to grow a piece from PF-3845 to mimic its function for binding without losing cellular activity. Alternatively, no attempt to displace the sulfate ion could be made in design because it appears readily available because of a good number of sulfate transporters and sulfatases in mycobacteria (38).

PF-3845 does not inhibit either human cytoplasmic or mitochondrial PheRS in biochemical assays at concentrations up to 200 μM (Figs. S6 and S13); the experimental data are supported by protein structural analysis. When comparing the catalytic site of Mtb PheRS with hFARS1 (39) and hFARS2 by structural superposition and sequence alignment, a major difference is found in the amino acid pocket (Fig. S14). Residues F438 in hFARS1 and M258 in hFARS2, which form the pocket at the same position, are much larger than the corresponding residue V286 in Mtb PheRS, resulting in the smaller amino acid pocket of hFARSs. Especially, F438 of hFARS1 reaches the position that could have steric clash with the thiazole ring of GDI05-001. GDI05-001 can therefore be effectively blocked from binding to human PheRSs. This analysis agrees with previous toxicity data (11, 15) and explains the selectivity and antibacterial potency of GDI05-001. Likewise, there is not enough space in hFARSs to accommodate PF-3845 since its two linked aromatic moieties take the same space as the thiazole ring of GDI05-001. Thus, the two hFARSs are not inhibited by PF-3845.

The cytotoxicity of PF-3845 against human cancer cell line has already been reported (40). In a previous medicinal chemistry program aiming at improving PF-3845 for the treatment of inflammatory pain and other nervous system disorders, the kinetic parameter *k*_inact_/*K*_i_ for covalent inhibition of human FAAH increased from 12,600 M^−1^s^−1^ (PF-3845) to 40,300 M^−1^s^−1^ (PF-004457845) (41). Our preliminary result showed that PF-004457845 with CC_50_ at 23.6 to 25.8 μM against two human cell lines is only slightly less toxic than PF-3845 with CC_50_ at 16.9 to 21.7 μM (Fig. S12). It indicates that cellular protein(s), other than the FAAH, are more potently inhibited by PF-3845 than PF-004457845. The unidentified proteins could be responsible for the viability of the human cells. More changes will need to be introduced into the PF-3845 scaffold when it is modified toward a TB drug lead. The robust ATP-dependent enzyme assays detailed in this work will assist the effort.

However, PF-3845 (PubChem CID: 25154867) has its own complexity. Its molecular weight of 456.5 g/mol and cLog*P* equivalent of 4.4 are at the upper limit of Pfizer's rule of five that evaluates the drug likeness of a compound based on orally active drugs for humans. A cautionary note is that the CC_50_ of PF-3845 to mammalian cells is even lower than its MIC against Mtb H37Rv, leaving it at a position unpropitious to become a TB drug candidate. Nevertheless, as tens of synthetic analogs of PF-3845 have been reported in the literatures (28, 42, 43), the PF-3845 scaffold can be used as an excellent chemical probe to study the function and evolution of bacterial and human PheRSs.

## Experimental procedures

### PheRS expression plasmids

The native sequences of Mtb *pheS* (National Center for Biotechnology Information gene ID: 885105) and *pheT* (National Center for Biotechnology Information gene ID: 885283) were synthesized by GenScript and cocloned into the NdeI and HindIII sites of pET-30a expression vector. Between the two ORFs, a ribosome-binding site (underlined) (…*pheS*…GGTGCCTAGTCTAGaaactaagaaggagatatacatATGGCCAGC…*pheT*…) was incorporated to increase the expression level of *pheT*. Only the C-terminal of PheT was designed to be fused to a His6 tag for copurification of the two subunits (Fig. S2*A*).

### Overexpression and purification of PheRS

The expression plasmid was transformed to BL21(DE3) competent cells, and transformants were selected by antibiotic marker. Single colony was inoculated into 220 ml LB medium containing 20 μg/ml kanamycin or 100 μg/ml ampicillin, and the culture was incubated for 16 h at 37 °C. A fresh culture of 9 ml was used to inoculate 1 l LB broth medium (total 24 l for one preparation) and grown at 37 °C until the absorbance at 600 nm reached 0.7 to 0.9. The culture was cooled down to 16 °C, and then IPTG was added to the final concentration of 0.3 mM. After 18 h of incubation at 16 °C, cells were harvested by using centrifuge (3800*g*, 15 min, 4 °C) and resuspended with cell harvesting buffer (25 mM Tris pH 8.0, 150 mM NaCl, 10 mM imidazole pH 8.0, 1 mM DTT, and EDTA-free protease inhibitor tablet [Roche]). Resuspended cells were divided into two portions and handled independently to lyse through cell disrupter (700–800 bar, five cycles) followed by 3-min sonication (60% amplitude). Cell debris was removed through centrifugation (24,000*g*, 80 min) followed by filtration against 0.22 μM polyvinylidene fluoride membrane (Millipore), and then the supernatant was loaded onto HisTrap-FF (GE Healthcare; 1 column volume [CV] = 5 ml, two columns in tandem). The flow rate was 2.5 ml/min. After washing 20 CV with washing buffer (25 mM Tris pH 8.0, 150 mM NaCl, 20 mM imidazole pH 8.0, and 1 mM DTT), fractions were collected with gradient elution buffer (25 mM Tris pH 8.0, 150 mM NaCl, 20–300 mM imidazole pH 8.0, and 1 mM DTT). Based on samples examined with SDS-PAGE and stained with Coomassie blue, the proper fractions were combined and applied to anion exchange purification with HiTrap Q-HP (GE Healthcare; 1 CV = 5 ml, two columns in tandem). After washing 12 CV with washing buffer (25 mM Tris pH 8.0, 10 mM NaCl, and 1 mM DTT), fractions were collected with gradient eluted buffer (25 mM Tris pH 8.0, 20–500 mM NaCl, and 1 mM DTT). Proper fractions based on SDS-PAGE analysis were collected and concentrated to about 2 ml and injected to Superdex-200 increase (flow rate: 0.3 ml/min, ∼1.4 Mpa; GE Healthcare) for further purification. The proper fractions were pooled together (25 mM Hepes pH 7.5, 150 mM NaCl, 5 mM MgCl_2_, and 5% glycerol) and concentrated to about 2 ml using Amicon-centrifugal filter (Millipore). UV_280_ absorbance of PheRS was measured on NanoDrop OneC (Thermo). Extinction coefficient of PheRS (*ɛ* = 111,840 M^−1^ cm^−1^) was calculated by entering alpha–beta heterodimer sequences into protparam program on ExPASy web server. PheRS used in nonradioactive assays was pooled with pure fractions of multiple rounds of preparation, and the protein concentration was calculated by Beer's law. It was dispensed into 50 μl aliquots and stored at −80 °C for biochemical assay and crystallization trials.

### tRNA expression plasmid

Sequence of Mtb-tRNA_TGG_^Phe^ gene with T7 promoter (underlined) at the 5′-terminal and CCA at the 3′-terminal (taatacgactcactataGGCCAGGTAGCTCAGTCGGTATGAGCGTCCGCCTGAAAAGCGGAAGGTCGGCGGTTCGATCCCGCCCCTGGCCAcca) was synthesized (GenScript). An EcoRI site was designed upstream of T7 promoter, and a BamHI site was downstream of the tRNA gene. The fragment was cloned into the EcoRI and BamHI sites of pTrc99a vector (Addgene).

### Overexpression and partial purification of tRNA^Phe^

The expression plasmid was transformed to BL21(DE3) competent cells. After incubation overnight on ampicillin containing LB agar, single colony was inoculated to 100 ml LB broth medium containing 100 μg/ml ampicillin and incubated for 16 h at 37 °C. A fresh culture of 10 ml was used to inoculate 1 liter LB broth medium (total 10 l for one preparation) and grown at 37 °C until the absorbance at 600 nm reached 0.6 to 0.8, then IPTG was added to final concentration of 0.4 mM. After 16 h incubation at 37 °C, cells were harvested by using centrifuge (3800*g*, 15 min, 4 °C) and resuspended in cell lysis buffer (100 mM Tris pH 7.0, 20 mM MgCl_2_; 30 ml per 1 l culture). Then equal volume of water-saturated phenol pH4 was added and fully mixed at room temperature for 1 h. After centrifugation (14,000*g*, 30 min, 25 °C), the upper aqueous layer was carefully transferred to be mixed with equal volume of chloroform. The mixing was gently performed, and the mixture was let stand for a while. After short centrifugation, carefully transfer the upper layer into multiple new tubes. Triple volumes of precold ethanol were added in, and nucleic acid was precipitated at −20 °C for more than 2 h. Supernatant was discarded after centrifugation (14,000*g*, 30 min, 4 °C). A pellet was dissolved in 10 ml deacylation buffer (500 mM Tris pH 9.0) and incubated at 37 °C for 1 h. Nucleic acid was afterward precipitated as before, and a pellet was washed two times with 75% precold ethanol by centrifugation, dried in air, and redissolved in 10 ml diethyl pyrocarbonate–treated water. The dissolved nucleic acid was mixed with 1 M MOPS pH 7.0 buffer to a final concentration of 0.1 mM. One Q-2500 column (Qiagen) (for every 2 l starting culture) was pre-equilibrated with 50 ml equilibration buffer twice (50 mM MOPS pH 7.0, 15% isopropanol, 1% Triton-X100). The nucleic acid was bound onto the column and washed four times with 50 ml washing buffer (50 mM MOPS pH 7.0, 200 mM NaCl) by gravity flow. Then, each column was washed with 10 ml elution buffer once (50 mM Mops pH 7.0, 650 mM NaCl), followed by at least 2 × 10 ml the same buffer that eluted mainly tRNAs. The elution fractions could be examined by Tris–borate–EDTA urea gel (Novex), and pure tRNA factions were precipitated by adding 1/10 volume of 3 M sodium acetate and equal volume of isopropanol for 2 h at −20 °C. The pellet was washed twice with 75% precold ethanol by centrifugation, air dried, and dissolved in tRNA storage buffer (50 mM HEPES pH 7.4, 20 mM MgCl_2_, 100 mM KCl) to final concentration of 10 mg/ml. It was dispensed to 100 μl aliquots and stored at −20 °C.

### PPi production assay

The assay was based on modification of EnzChek Phosphate assay kit (Thermo). Several aaRS works reported using this assay (3, 26, 27, 44). Our assay was performed in Corning 384-well flat clear bottom black microplate (catalog no. 3764), and the reaction plates were read on EnVision (PerkinElmer). The final concentration of each component in the reaction buffer is 50 mM Hepes pH 7.4, 100 mM NaCl, 10 mM MgCl_2_, 1 mM tris(2-carboxyethyl)phosphine, 0.1 mg/ml bovine serum albumin, 0.01% BriJ-35 (Sigma). In IC_50_ determination, the reaction was started with 20 μl volume in a well containing 50 nM Mtb PheRS, 0.5 mM ATP, 50 μM l-Phe, 0.1 mg/ml tRNA^Phe^, 0.05 mM MESG, 0.5 μ/ml PPase (Sigma), and 0.1 μ/ml PNPase (Thermo). Absorbance at 360 nm was read every minute for 30 min. PPi standard curve was made with 0.05 mM MESG, 0.5 μ/ml PPase, 0.1 μ/ml PNPase, and 1.25 to 20 μM sodium PPi, and the reactions were incubated for 15 min before read at 360 nm. Readout was converted to PPi production using the curve. GraphPad Prism 9 was used for data analysis.

### ATP consumption assay and HTS

This assay used for HTS was based on Kinase-Glo luminescent Kit (Promega). The application with aaRS was previously descried (27, 45). Screening was carried out in Corning 384-well white flat bottom microplates (catalog 3570). The final concentrations in the reaction are 25 mM Hepes pH 7.4, 140 mM NaCl, 40 mM MgCl_2_, 30 mM KCl, 1 mM tris(2-carboxyethyl)phosphine, 0.1 mg/ml bovine serum albumin, and 0.004% Tween-20. Compounds from Selleck-2148 bioactive library (Selleckchem) 10 mM stock were prepared in assay plate using Echo 550 (Labcyte) to final concentration of 50 μM. The 50-nL compound volume is negligible in each reaction. Dimethyl sulfoxide (DMSO) was added into negative control wells, and tool compound GDI05-001 was added in positive control wells. About 5 μl Mtb PheRS in Hepes buffer was added to 100 nM using Multidrop dispensers (Thermo), and the compound–enzyme mixture was incubated for 30 min. Then 5-μl substrate mixture (1 μM ATP, 20 μM l-Phe, and 0.1 mg/ml tRNA^Phe^) was added to start the reaction at 37 °C and incubated for 2 h. After cooling down the plates, 10-μl diluted Kinase-Glo Max reagent (1/50 diluted with buffer 50 mM Tris pH 7.5, 5% glycerol) was added and incubated for 15 min. Luminescence was recorded using SpectraMax M5 (Molecular Devices).

### IC50 determination

Compound was prepared using Echo 550 in 384-well plates in a series of 10 concentrations. DMSO wells were of negative control, and no enzyme wells were of positive control. For assay with Mtb PheRS, the same method was used as aforementioned. For hFARS1, 50 nM enzyme, 2 μM ATP, 20 μM l-Phe, and 1 mg/ml yeast total tRNA (Roche) were incubated with compound at 37 °C for 1 h. For hFARS2, the same method as hFARS1 except that *E. coli* total tRNA (Roche) was used at 0.5 mg/ml. Plates were processed for luminescence as aforementioned. In data analysis with the GraphPad Prism, the enzyme remaining activity was calculated as:

$$\text{Activity}\;\left(\text{\%}\right)\;=\;\left(1\;-\;\frac{{\text{Lum}}^{max}\;-\;{\text{Lum}}^{I}}{{\text{Lum}}^{max}\;-\;{\text{Lum}}^{min}}\right)\;\times \;100$$

Lum^*max*^ was luminescence value without PheRS in reaction, Lum^*min*^ was the value from DMSO control well, and Lum^*I*^ was from inhibitor well. The activity (percentage) was plotted against inhibitor concentration. To calculate IC50, the resulted dose–response curve was fit to Equation 1, where H was the Hillslope factor.

(1) $$y\;=\;y_{min}\;+\;\frac{{\text{y}}_{max}\;\pm \;{\text{y}}_{min}}{1\;+\;{\left(\frac{IC_{50}}{x}\right)}^{H}}$$

### Mode of inhibition

By the PPi production assay, 50 nM of Mtb PheRS with two saturating substrates and one variable substrate were used in the measurement of steady-state enzyme kinetics at a series of fixed concentrations of PF-3845 (MCE MedChemExpress). Lineweaver–Burk plots were fitted for modeling of inhibition (competitive, noncompetitive, mixed, or uncompetitive). To calculate the *K*_i_ for competitive inhibition, the plots with PPi production rate against substrate concentration were fit to:

(2) $$v_{0}\;=\;\frac{{\text{V}}_{max}\left[\text{S}\right]}{K_{M}\left(1+\frac{\left[I\right]}{K_{i}}\right)\;+\;\left[S\right]}$$

To calculate the *K*_i_ for mixed inhibition, the plots were fit to:

(3) $$v_{0}\;=\;\frac{{\text{V}}_{max}\left[\text{S}\right]}{K_{M}\left(1\;+\;\frac{\left[I\right]}{K_{i}}\right)\;+\;\left[S\right]\left(1\;+\;\frac{\left[I\right]}{\alpha K_{i}}\right)}$$

### Crystallization, data collection, and structure determination

Mtb PheRS was incubated with GDI05-001 or PF3845 at a molar ratio of 1:5 for 4 h. About 5 mg/ml of the apo form and two complexes were crystallized separately by sitting drop vapor diffusion method at 18 °C. The best crystals were obtained by seeding with well buffer containing 0.1 M Hepes pH 7.7, 1.5 M (NH_4_)_2_SO_4_, 0.2% PEG 3350. The cryoprotectant solution contained 0.1 M Hepes pH 7.7, 1.5 M (NH_4_)_2_SO_4_, 0.2% PEG 3350, and 25% glycerol. X-ray data were collected in Shanghai Synchrotron Radiation Facility at 100 K. Data integration and scaling were performed using HKL2000. These crystals belong to the space group C2221 and contain one PheRS heterodimer per asymmetric unit. The apo form structure was determined by molecular replacement with the Phaser-MR module in Phenix using *E. coli* PheRS (Protein Data Bank code: 3PCO) as a search model. The sequence identity between the α subunits of Mtb and *E. coli* PheRS is 46.8% with the r.m.s.d of 1.397 Å, whereas that between the β subunits is 32.1% with the r.m.s.d of 2.466 Å. The model adjustment and refinement were performed using COOT and Phenix. Structures of the two liganded TbPheRS complexes were determined using the coordinate of the apo form structure. The final refinement statistics are summarized in Table 1. All figures were prepared using Pymol. The relevant coordinates and structure factors have been deposited in Protein Data Bank with accession codes 7DAW, 7DB7, and 7DB8.

**Table 1:** Data collection, phasing, and refinement statistics

Data collection	Mtb PheRS (apo form)	Mtb PheRS–GDI05-001	Mtb PheRS–PF-3845
Space group	C2221	C2221	C2221
Resolution (Å)^a^	50.0–2.83 (2.83–2.87)	50.0–2.71 (2.71–2.75)	50.0–2.30 (2.30–2.34)
Cell dimensions			
a, b, c (Å)	267.00, 298.91, 65.94	268.38, 298.80, 65.71	278.01, 297.81, 65.90
α, β, γ (°)	90, 90, 90	90, 90, 90	90, 90, 90
Unique reflections	59,426 (2275)	72,740 (3594)	121,928 (6050)
I/σI	10.86 (1.67)	10.42 (3.26)	19.0 (4.1)
Completeness (%)	93.5 (71.5)	99.9 (100.0)	99.9 (100.0)
R_merge_ (%)	17.4 (45.5)	13.3 (76.6)	13.8 (68.1)
CC1/2 (%)	96.4 (84.0)	98.5 (85.1)	99.4 (95.3)
Wilson B factor (Å^2^)	59.36	45.37	29.83
Refinement			
R_work_ (%)	20.00	19.20	16.83
R_free_(%)	24.02	21.76	18.65
Average B factors (Å^2^)	61.02	43.58	35.26
R.M.S.D.			
Bond lengths (Å)	0.009	0.009	0.008
Bond angles (°)	1.109	1.11	0.997
Ramachandran plot			
Favored (%)	94.26	95.27	96.99
Allowed (%)	5.47	4.37	2.91
Outliers (%)	0.27	0.36	0.09

a Numbers in parentheses are for the highest resolution shell.

### Cytotoxicity measurement

HEK293T, Vero E6, and HepG2 cells were cultured in Dulbecco's modified Eagle's medium plus 10% fetal bovine serum and 1% penicillin/streptomycin at 37 °C with 5% CO_2_. Plates of 96 wells were plated with 100 μl 1 × 10^5^ cells/ml per well and incubated for 24 h before adding compound in serial dilutions. After another 48 h, 40 μl reagents of CellTiter-Glo cell viability assay (Promega) was added, and the plates were shaken at 400 rpm for 10 min in the dark. Luminescence was recorded with plate reader. The data were analyzed with GraphPad Prism 9.

### Susceptibility of WT H37Rv and pheS–FDAS to PF-3845

The Mtb strains were grown in 7H9 broth (supplemented with 5 g/l bovine serum albumin, 2 g/l dextrose, 0.85 g/l NaCl, 0.2% glycerol, and 0.05% tyloxapol, and +50 μg/ml hygromycin for pheS–FDAS) in a humidified incubator at 37 °C and 5% CO_2_ until logarithmic growth was reached. The cultures were washed with fresh drug-free 7H9 and suspended to an absorbance at 580 nm of 0.01. The diluted suspensions were added to 384-well black and clear-bottom plates at 50 μl/well, containing 14-point twofold drug dilutions in DMSO in triplicate, for a final DMSO concentration of 1%. Plates were wrapped with aluminum foil in stacks of three and incubated at 37 °C and 5% CO_2_ in a humidified incubator. The absorbance at 580 nm of the plates was read after incubating 11 days. Growth data were normalized to inhibitor-free wells, and MIC values were determined by fitting the normalized data to a Gompertz model in GraphPad Prism 8.

## Data availability

This study contains supplementary Figures S1–S14. Protein structures and the relevant coordinates have been deposited in Protein Data Bank with accession codes 7DAW, 7DB7, and 7DB8.

## Conflict of interest

The authors declare that they have no conflicts of interest with the contents of this article.
