# Supplementary material for: Rediscovery of PF-3845 as a new chemical scaffold inhibiting phenylalanyl-tRNA synthetase in Mycobacterium tuberculosis
Source: J Biol Chem. 2021 Jan 8;296:100257. doi: 10.1016/j.jbc.2021.100257 (PMC7948948; doi:10.1016/j.jbc.2021.100257)
Supplement: Supplementary file 1 — Figures S1 to S14 [file mmc1.pdf]

Supporting Information

Re-discovery of PF-3845 as a new chemical scaffold inhibiting phenylalanyl-tRNA synthetase in *Mycobacterium tuberculosis*

Heng Wang[1]*, Min Xu[1]*, Curtis A Engelhart[2], Xi Zhang[1], Baohua Yan[3], Miaomiao Pan[1], Yuanyuan Xu[1], Shilong Fan[3], Renhe Liu[1], Lan Xu[1], Lan Hua[1], Dirk Schnappinger[2], Shawn Chen[1]**

[1]Global Health Drug Discovery Institute, Haidian, Beijing, China

[2]Department of Microbiology and Immunology, Weill Cornell Medical College, New York, New York, USA

[3]Center of Protein Science Facility, Tsinghua University, Beijing, China

*These authors contributed equally to this work.

**Corresponding author: Shawn Chen

E-mail: schen169@hotmail.com or shuo.chen@ghddi.org

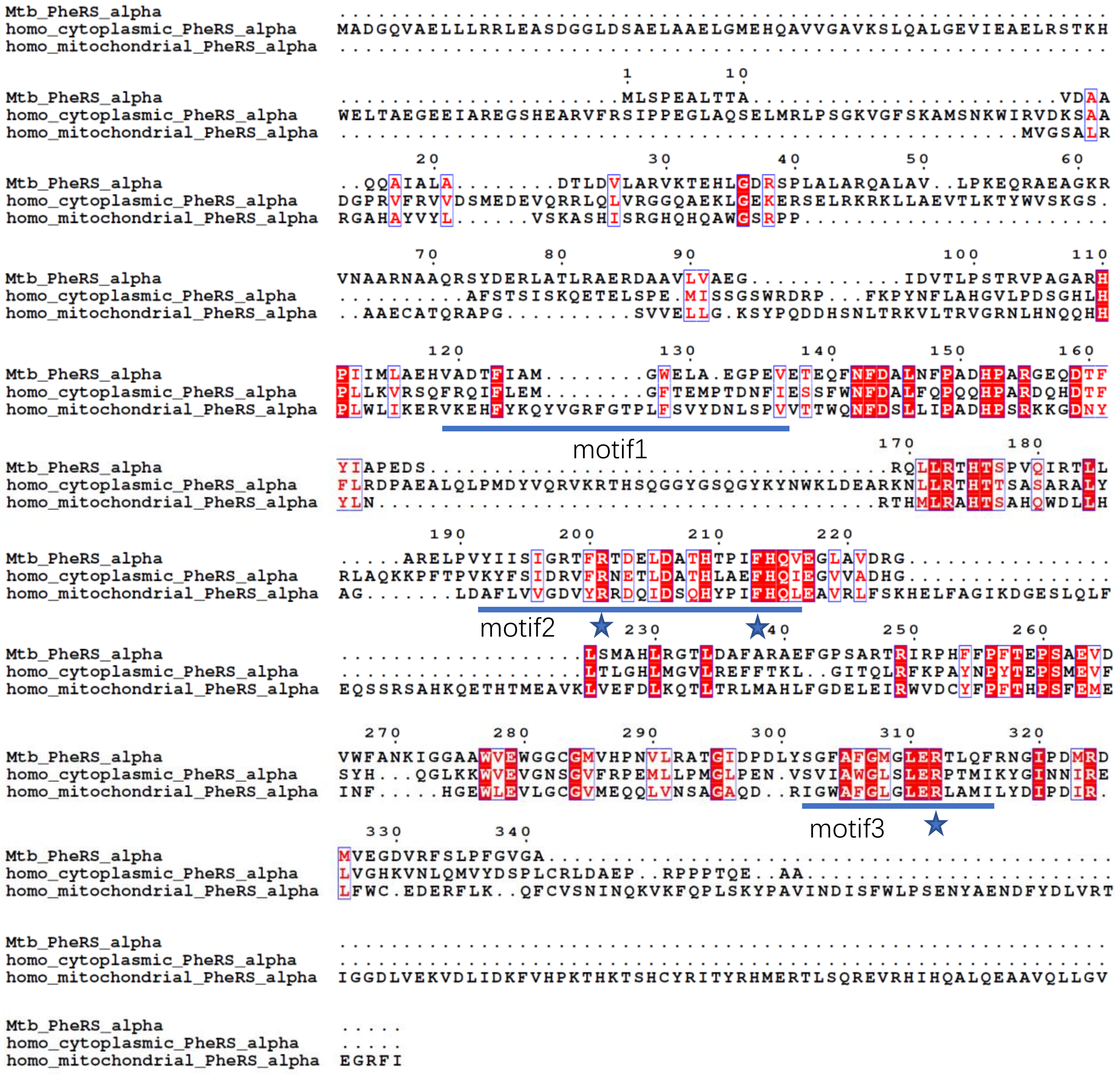

Figure S1. Alignment of PheRS α subunits and identification of motifs. Motif 1 forms part of the dimer interface and helps orient the motif 2 and 3 active site loops. Motif 2 participates in the binding of ATP, amino acid substrate, and the acceptor end of tRNA, while motif 3 interacts with ATP substrate. The highly conserved arginine and phenylalanine of motif 2, and the arginine of motif 3 marked by asterisks have catalytic roles.

A.

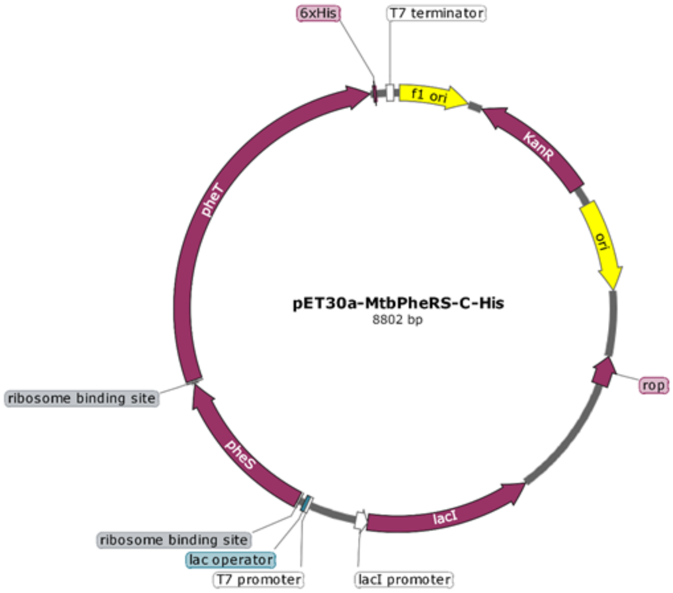

B.

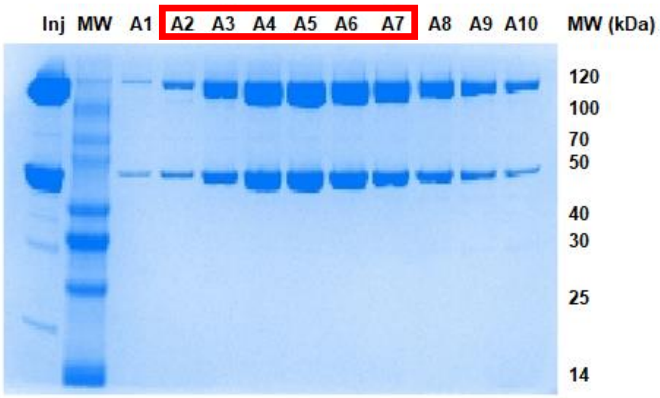

Figure S2. Plasmid map for PheRS overexpression in *E. coli* (A) and the purified PheRS shown by SDS-PAGE electrophoresis (B). The fractions (A1- A10) were from the last-step size exclusion column. Inj, sample before injecting into the column; MW, protein marker (Blue Plus® II, Transgen). Red box indicates the collected fractions of PheRS.

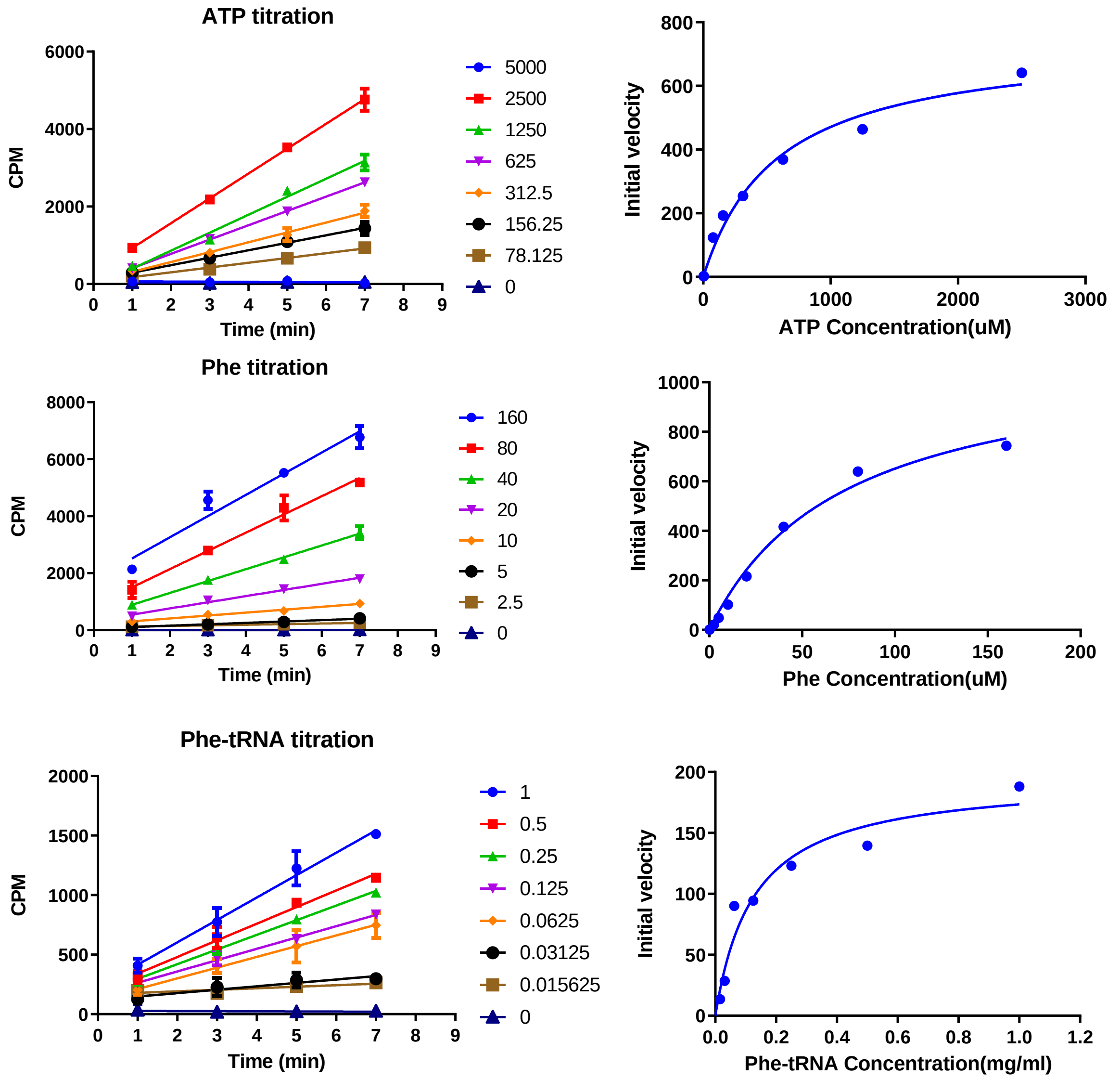

	ATP	Phe	*$tRNA^{Phe}$
K_m (μM)	387±279	68.0±6.6	2.0±0.6
k_{cat} (min^{-1})	24.3±25.0	66.3±3.1	12.8±4.4
k_{cat}/K_m (min^{-1} μM^{-1})	0.062	0.975	6.305

* $tRNA^{Phe}$ effective concentration was determined by measuring the counts of ^{14}C-Phe charged to saturation. 1 mg/ml = ~12.3 μM.

Figure S3. Michaelis-Menten kinetic parameters of Mtb PheRS determined by the aminoacylation assay. It is based on retention of L-[^{14}C(U)]-phenylalanine charged tRNA on GF/B fiberglass filter.

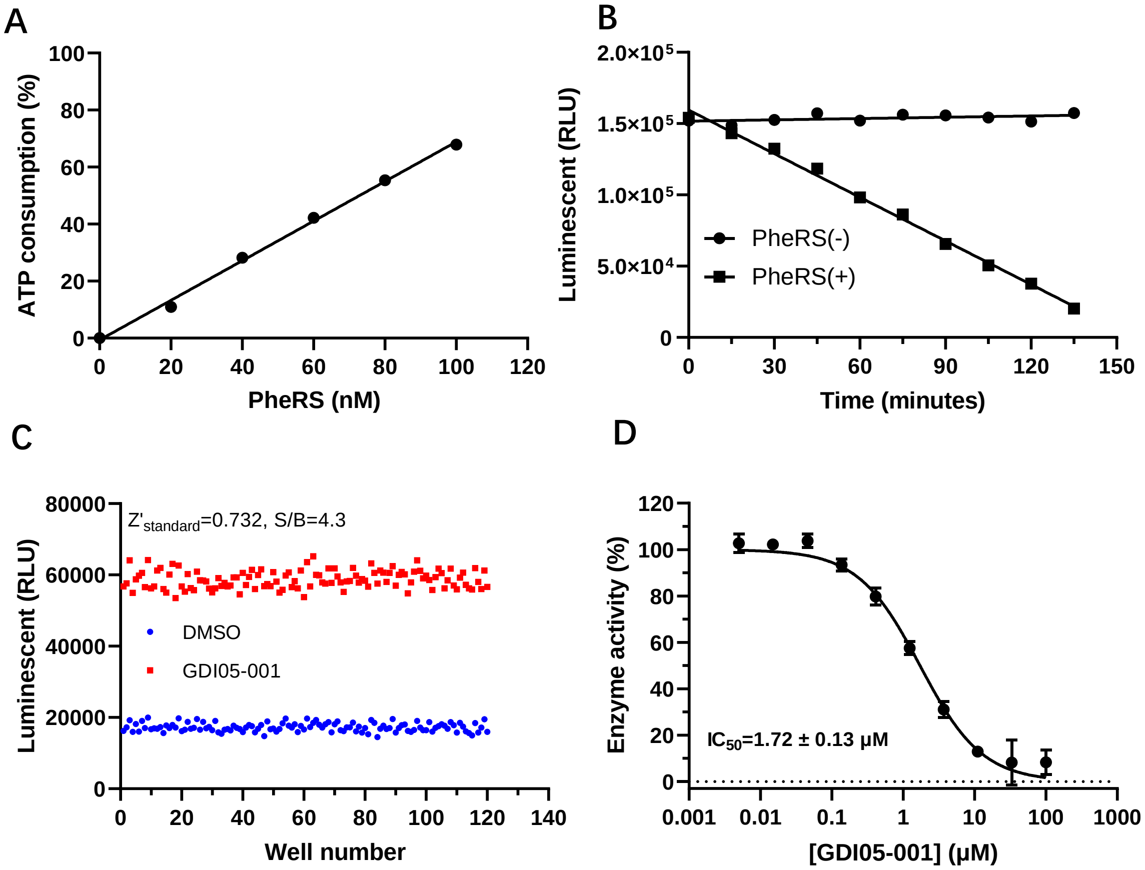

Figure S4. Development of HTS using ATP consumption assay (Kinase-Glo) and HTS optimization. A, Linearity of ATP consumption with respect to Mtb PheRS concentration. B, Linearity with respect to time with or without Mtb PheRS. C, Z' factor of HTS by using ATP consumption assay. D, IC_{50} of tool compound GDI05-001 against Mtb PheRS.

PF-3845 inhibition: 94.4%

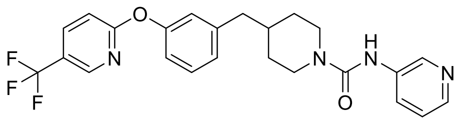

Toltrazuril inhibition: 93.9%

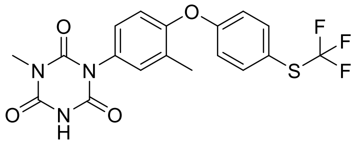

Zinc Pyrithione inhibition: 78.1%

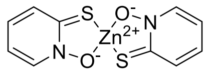

BMS-754807 inhibition: 76.5%

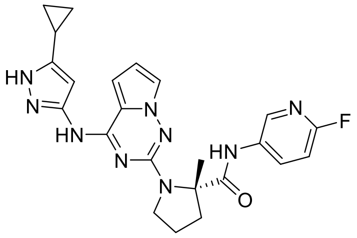

Figure S5. Primary hits of the Mtb PheRS HTS.

	IC$_{50}$ (μM)					
	Kinase Glo, Mtb PheRS	PPi Product, Mtb PheRS	AMP Glo, Mtb PheRS	Malachite Green, Mtb PheRS	Kinase Glo, hFARS1	Kinase Glo, hFARS2
GDI05-001	1.72±0.13	1.34	0.87±0.06	0.52±0.06	>200	85.44
PF-3845	2.65±0.19	2.40	2.49±0.19	1.95±0.19	>200	>200
PF-04457845	9.76±0.73	12.05	10.14±1.02	11.69±1.43	>200	>200

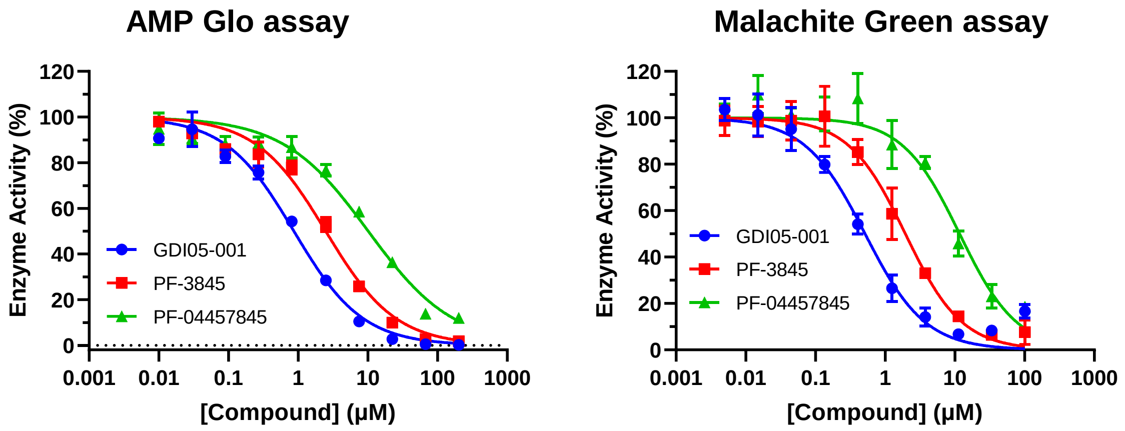

Figure S6. IC$_{50}$s of compounds against Mtb PheRS or hFARSx determined by different other assays. Kinase-Glo and PPi production assays are mentioned in the text. AMP-Glo assay measures AMP production with an AMP-Glo kit (Promega). Malachite Green assay is an end-point assay measuring Pi production, previously applied to aaRS (ref. 29).

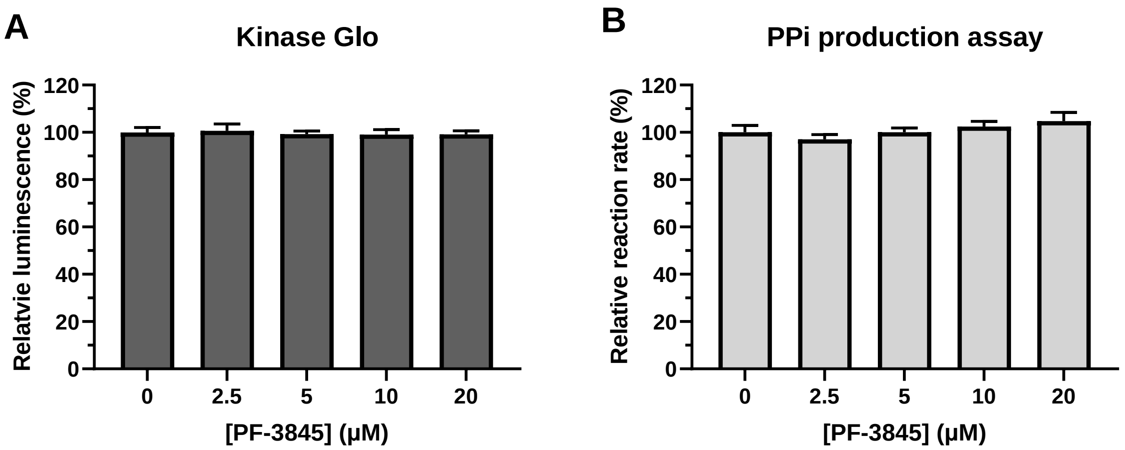

Figure S7. PF-3845 did not inhibit the coupled reactions in Kinase-Glo (A) and PPi production (B) assays. The two assay reactions were set up and monitored as those in IC$_{50}$ determination except that Mtb PheRS was omitted.

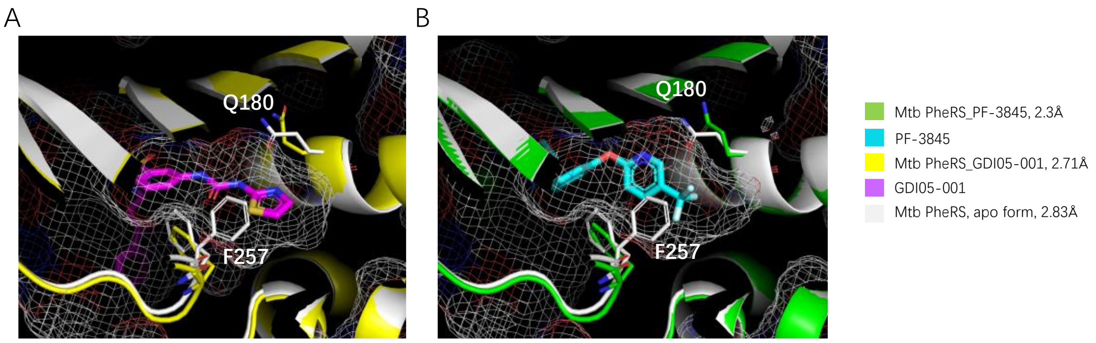

Figure S8. F257 and Q180 rotation creates a deeper amino acid pocket of Mtb PheRS. The side chain rotation of F257 and Q180 contributes to a much deeper amino acid pocket in the complexed structure than that in apo form structure. The amino acid pockets are indicated by mesh. A. Superposition of apo and GDI05-001 complexed structures. B. Apo and PF-3845 complexed structures.

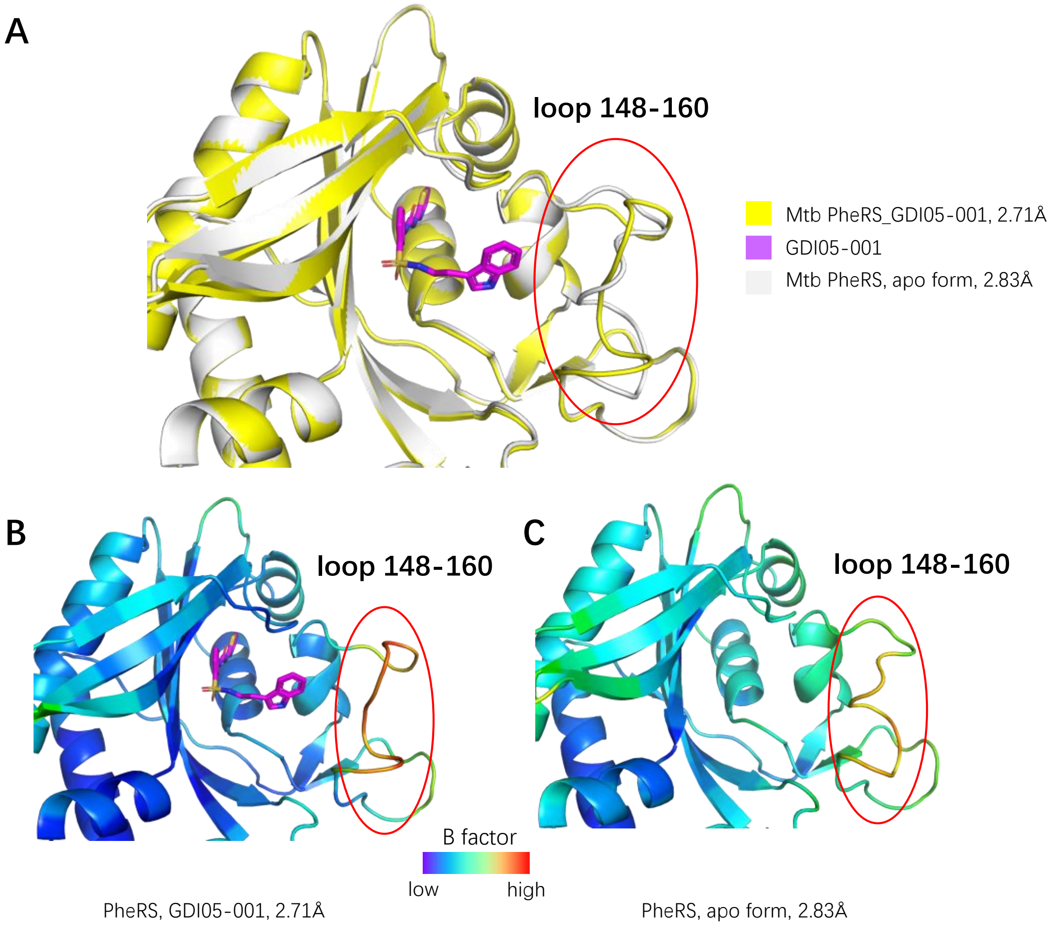

Figure S9. GDI05-001 binding leads to conformational change of the loop 148-160 of α subunit of Mtb PheRS. A, structure comparison of the α subunits of Mtb PheRS apo form and in complex with GDI05-001. B-C, structure colored by B factors. Compared to apo structure, the loop 148-160 of the GDI05-001 bound structure undergoes a conformational change, which has much higher B factors than the apo structure.

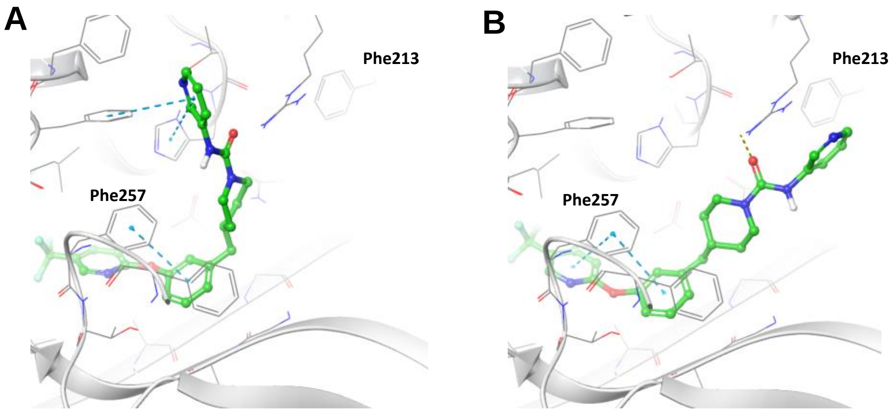

Figure S10. Two putative binding conformations of PF-3845 generated by Orbital docking platform (Accutar Biotech). A, PF-3845 is modelled into the substrate Phe and additional pockets. B, PF-3845 is modelled into both Phe and ATP binding pockets.

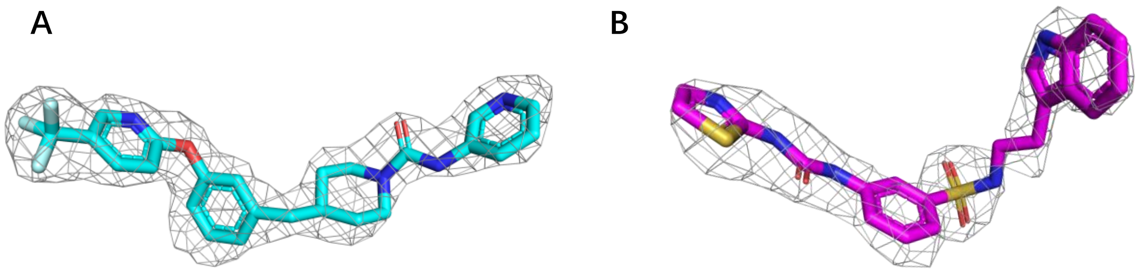

PF-3845, initial Fo-Fc map from the molecular replacement phasing, contour level= 3.0

GDI05-001, initial Fo-Fc map from the molecular replacement phasing, contour level= 3.0

Figure S11. Initial Fo-Fc maps of PF-3845 (A) and GDI05-001 (B) from the molecular replacement phasing.

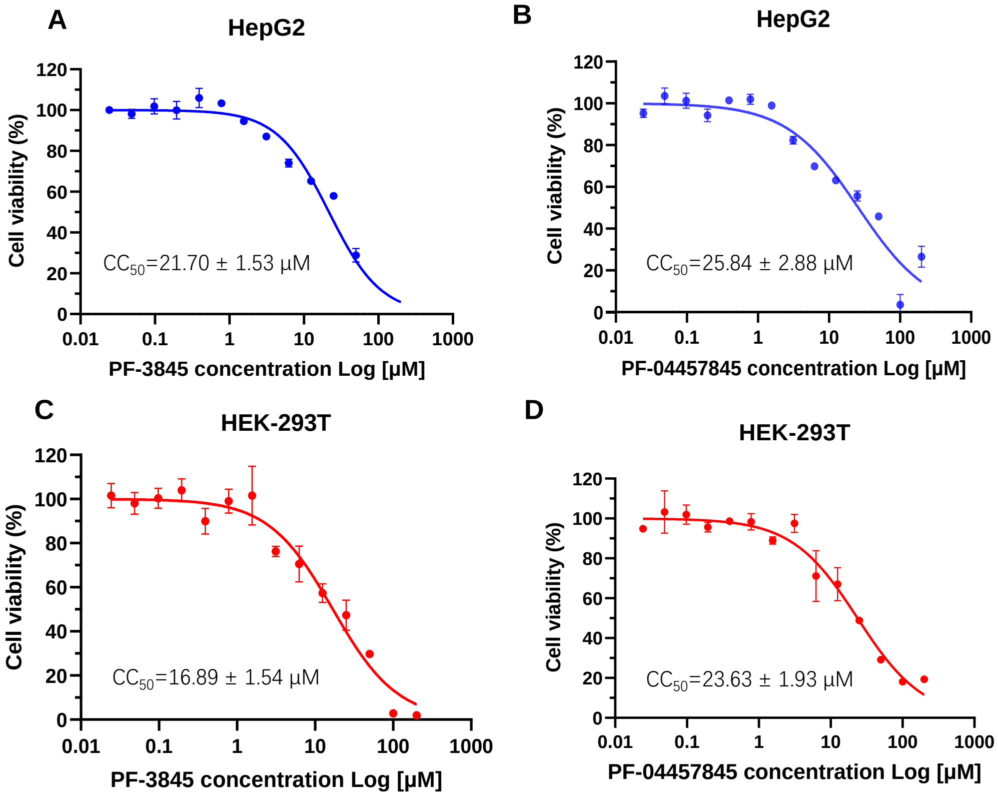

Cytotoxicity (μM)	PF-3845	PF-04457845
**HepG2**	21.7	25.8
**HEK-293T**	16.9	23.6
**Vero E6**	21.7	--

Figure S12. Cytotoxicity of PF-3845 and PF-004457845 against mammalian cell lines. HepG2 (A and B) is an immortal cell line derived from human liver cells. HEK-293T (C and D) is a human embryonic kidney 293 cell line. Vero E6 is a cell line derived from African green monkey kidney epithelial cells.

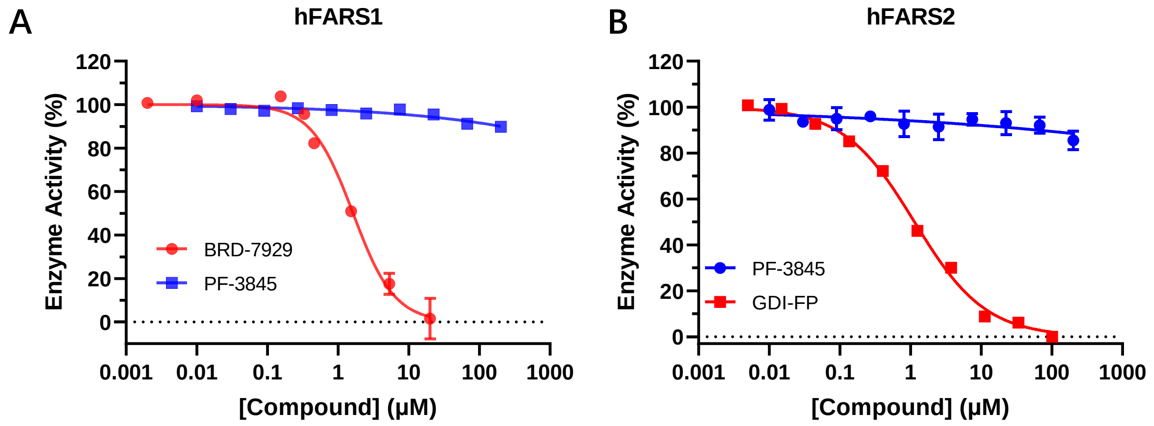

Figure S13. Analysis of potential effect of PF-3845 against human PheRS, cytoplasmic hFARS1 (A) or mitochondrial hFARS2 (B). Compound BRD-7929, known to have a level of inhibitory effect on eukaryotic PheRS (ref. 20), was used as positive control in the hFARS1 experiment. Compound GDI-FP undisclosed was used as positive control in the hFARS2 experiment. The same strategy for cloning Mtb PheRS was used to synthesize and construct expression plasmid for human cytoplasmic PheRS (hFARS1, Gene IDs 2193 and 10056). Gene for human mitochondrial PheRS (hFARS2, Gene ID 10667) did not include mitochondria signal peptide sequences (37 amino acids at the N-terminal), and was cloned into the NdeI and SalI sites of pET-21a vector, which gives a fusion protein with C-terminal His6-tag. Genes for the two human PheRSs were codon optimized for heterologous expression in *E. coli*. The protein purification steps were very similar to Mtb PheRS.

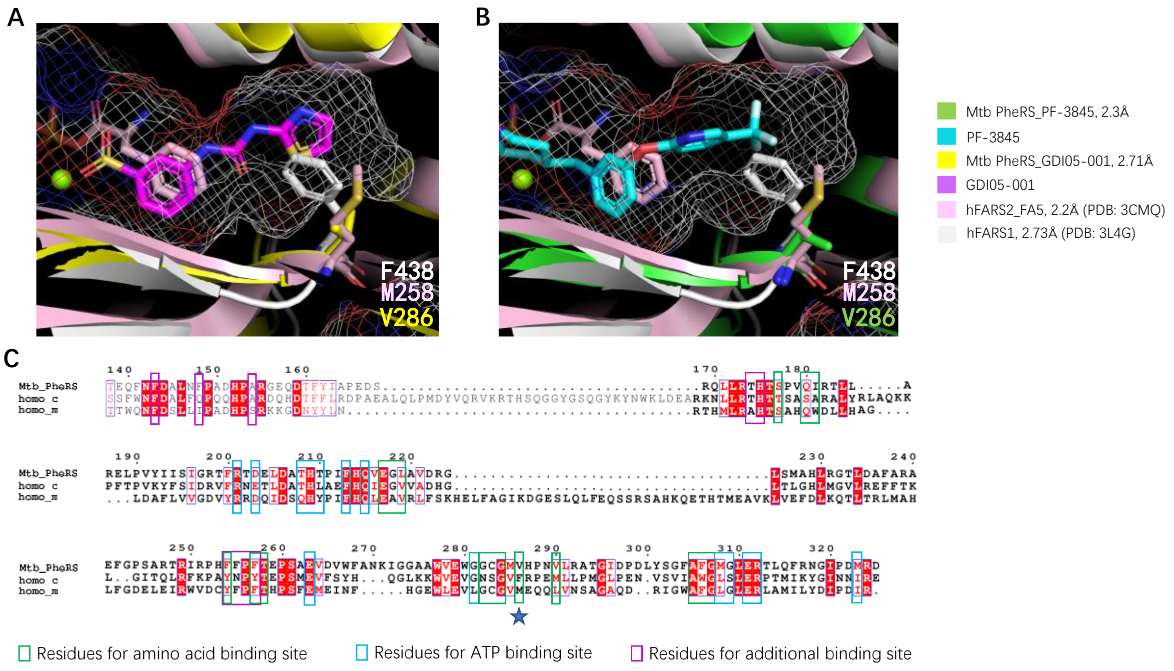

Figure S14. The major difference between the amino acid pockets of Mtb and human PheRSs. A, structure superposition of the amino acid pockets of MtbPheRS-GDI05-001, hFARS1 apo form and hFARS2-Phe-AMP. V286 in Mtb PheRS, F438 in hFARS1 and M258 in hFARS2 are shown in stick. The amino acid pocket of MtbPheRS-GDI05-001 are indicated by mesh. B, structure superposition of the amino acid pockets of MtbPheRS-PF-3845, hFARS1 apo form and hFARS2-Phe-AMP. V286 in Mtb PheRS, F438 in hFARS1 and M258 in hFARS2 are shown in stick. The amino acid pocket of MtbPheRS-PF-3845 are indicated by mesh. C, sequence alignment between Mtb and human PheRS. Key residues forming the three pockets are indicated by boxes in different colors. The most important residue that contributes to the difference of amino acid pocket between Mtb and human is indicated by blue star.

Interpretation of GDI05-001 NMR and mass spectrum

The ^{1}H spectra were recorded in MeOD on Bruker-400 NMR spectrometer. Mass spectrum was collected on Waters UPLC with QDa detector.

^{1}H NMR (400 MHz, MeOD) d ppm 2.88 (t, J=7.2 Hz, 2H), 3.20 (t, J=7.6 Hz, 2H), 6.94–7.05 (m, 4H), 7.36 (d, J=3.6 Hz, 1 H), 7.39–7.48 (m, 4H), 7.64 (d, J=3.6 Hz, 1 H), 8.06 (s, 1 H); MS (ESI) m/z calculated for $C_{20}H_{19}N_5O_3S_2$ $[M + H]^+$ 442.1, found 442.3.
